# A conceptual framework for LGBTQ+ consumer decision-making across health, housing, financial, and service areas

**DOI:** 10.3389/fpsyg.2026.1841095

**Published:** 2026-09-10

**Authors:** Peter G. Kreysa

**Affiliations:** California State University, Long Beach, CA, United States

**Keywords:** affirming cues, consumer behavior, decision-making, discrimination, LGBTQ+, risk perception, trust

## Abstract

**Purpose:**

LGBTQ+ consumers routinely navigate service environments shaped by longstanding inequities, uneven legal protections, and assumptions that fail to reflect their lived experiences. Although research on LGBTQ+ health disparities has expanded, far less is known about how LGBTQ+ individuals make decisions as consumers in areas, such as housing, financial services, and everyday service interactions. This article presents a conceptual framework that integrates these areas by examining how trust, risk perception, discrimination history, and affirming cues jointly shape LGBTQ+ consumer decision-making.

**Methods:**

This conceptual analysis synthesizes interdisciplinary literature from consumer behavior, public health, social psychology, and LGBTQ+ studies. The framework draws on empirical findings related to minority stress, service discrimination, and identity-based decision processes to explain how structural and interpersonal factors influence consumer judgments across multiple service contexts.

**Results:**

The framework identifies four interconnected constructs that include trust, risk perception, discrimination history, and affirming cues. These constructs guide LGBTQ+ consumers’ assessments of service environments and interact to influence how individuals interpret provider behavior, anticipate potential harm, and decide whether, when, and how to engage with services. The analysis suggests that LGBTQ+ consumers often rely on heightened vigilance and identity-based filtering when navigating markets, particularly in settings where protections are weak or past discrimination is salient. These dynamics shape access to essential resources and contribute to disparities in service use and well-being.

**Conclusion:**

By integrating structural conditions with interpersonal experiences, this framework offers a foundation for future empirical research and highlights actionable steps that service providers and policymakers can take to create more inclusive, trustworthy, and equitable consumer environments.

## Introduction

Lesbian, gay, bisexual, transgender, queer, and other sexual and gender minority (LGBTQ+) people participate in the same consumer markets as everyone else; however, the conditions under which they do so are often quite different. Many service systems, whether in health care, housing, finance, or everyday retail, were built around assumptions that privilege heterosexual and cisgender experiences (Hatzenbuehler, 2010; Meyer, 2003). As a result, LGBTQ+ consumers frequently encounter environments in which policies, staff training, and institutional norms do not fully account for their identities or needs (Center for American Progress, 2025; Lambda Legal, 2010). Even routine interactions can carry the possibility of misgendering, privacy violations, biased treatment, or exclusion (Gonzales et al., 2016; Kaiser Family Foundation, 2024). For example, when LGBTQ+ individuals seek health care, apply for housing, open a bank account, or navigate a store, they must weigh not only the usual considerations of cost and quality but also the likelihood of discrimination, the presence of affirming cues, and the potential risks involved (Casey et al., 2019; Durso and Gates, 2012). These additional layers of assessment reflect a broader history of structural marginalization, inconsistent legal protections, and uneven enforcement of nondiscrimination policies (Human Rights Campaign Foundation, 2024; Romero et al., 2020).

These dynamics shape LGBTQ+ consumer behavior in ways that differ from the experiences of heterosexual and cisgender consumers whose identities are typically assumed and validated within most service contexts. Minority stress theory helps explain why stigma, prejudice, and discrimination create a social environment that can undermine LGBTQ+ people’s well-being and influence how they interact with institutions (Hatzenbuehler, 2010; Meyer, 2003). These stressors do not simply affect health outcomes. They also shape expectations, perceptions, and decision-making processes across a wide range of consumer settings.

Despite the relevance of these issues, research on LGBTQ+ consumer behaviors remains limited. Much of the existing scholarship focuses on health disparities, mental health, or discrimination in specific institutional settings, such as employment or health care (Fredriksen-Goldsen et al., 2013; Hatzenbuehler, 2010). Far less attention has been paid to LGBTQ+ individuals as consumers whose decisions are shaped by identity-specific concerns, historical marginalization, and the need to navigate environments that may or may not be affirming. At the same time, national surveys and policy reports continue to document persistent discrimination in housing, financial services, and access to basic resources.

This article addresses this gap by proposing a conceptual framework that explains how LGBTQ+ consumers make decisions across multiple areas. The following sections describe the broader social context shaping LGBTQ+ consumer experiences, introduce the four constructs that anchor the framework, and discuss the implications for service providers, policymakers, and researchers.

### LGBTQ+ consumers and the social context of decision-making

LGBTQ+ consumers move through service environments shaped by broader social forces that often place them at a disadvantage. Minority stress theory offers a useful starting point for understanding these dynamics. For example, the theory highlights how external stressors such as discrimination, harassment, and violence combine with internal stressors such as concealment or internalized stigma to influence LGBTQ+ people’s expectations and experiences in institutional settings (Hatzenbuehler, 2010; Meyer, 2003). These pressures not only affect mental or physical health; they also shape how LGBTQ+ individuals approach everyday interactions with service providers. Many enter such encounters with a heightened sense of vigilance, informed by past experiences of, say, rejection, misgendering, or exclusion in health care, housing, financial institutions, and other consumer contexts (Kcomt, 2019; Puckett et al., 2018).

Another way to understand these dynamics is through the lens of consumer vulnerability. For example, vulnerability arises when individuals are more susceptible to harm from personal, social, or environmental factors (Durso and Gates, 2012; Human Rights Campaign Foundation, 2024). For LGBTQ+ consumers, vulnerability may stem from identity-based stigma, economic instability, housing insecurity, or limited access to affirming services (Romero et al., 2020). National surveys consistently show that LGBTQ+ people experience higher rates of poverty, housing instability, and homelessness than their non-LGBTQ+ peers, and that they face significant barriers to securing safe and affordable housing (Center for American Progress, 2025; Romero et al., 2020). Similar patterns appear in health care, in which LGBTQ+ adults report high levels of discrimination and difficulty in accessing respectful, competent care (Kaiser Family Foundation, 2024).

Intersectionality adds another layer of complexity. For example, LGBTQ+ individuals who are also immigrants, people with disabilities, members of racial or ethnic minority groups, or individuals with low socioeconomic status often face overlapping forms of discrimination (Fredriksen-Goldsen et al., 2013; Hatzenbuehler, 2010). These intersecting identities can intensify vulnerability and shape consumer behavior in distinct ways, thereby influencing how individuals assess risk, anticipate treatment, and decide whether to engage with particular institutions.

Taken together, these contextual factors create the backdrop against which LGBTQ+ consumers make decisions. They also set the stage for the four constructs (trust, risk perception, discrimination history, and affirming cues) that form the core of the conceptual framework developed in the following section.

## Framework

The proposed framework is grounded in two complementary perspectives: Minority Stress Theory and Intersectionality Theory. Minority Stress Theory posits that sexual and gender minority individuals experience unique, chronic stressors related to stigma, prejudice, and discrimination that extend beyond everyday life stressors (Meyer, 2003; Hatzenbuehler, 2010). These stressors include both external experiences, such as discrimination and victimization, and internal processes, such as vigilance, identity concealment, and expectations of rejection. Although the theory was originally developed to explain mental health disparities, its core principles are also useful for understanding consumer decision-making because prior experiences with stigma and discrimination influence how LGBTQ+ individuals evaluate service environments, assess trustworthiness, and anticipate potential risks. Minority Stress Theory continues to guide contemporary LGBTQ+ research and has been applied across a growing range of health, social, and community contexts. Recent scholarship has further emphasized the importance of understanding how expectations of rejection, vigilance, and prior experiences of discrimination affect interactions with institutions and service providers. These insights support the application of Minority Stress Theory to consumer decision-making by highlighting how social experiences shape perceptions of trust, safety, and risk.

Intersectionality Theory complements Minority Stress Theory by recognizing that individuals possess multiple social identities that interact to shape experiences of privilege and disadvantage. LGBTQ+ individuals who are also members of racial and ethnic minority groups, have disabilities, are immigrants, or occupy other marginalized statuses may encounter overlapping forms of discrimination that influence consumer behavior in unique ways (Fredriksen-Goldsen et al., 2013). An intersectional perspective helps explain why LGBTQ+ consumers are not a homogeneous group and why trust, risk perception, and responses to affirming cues may differ substantially across individuals and contexts. Together, these perspectives provide the theoretical foundation for the proposed framework by linking structural inequities, lived experiences, and consumer decision-making processes.

Understanding how LGBTQ+ consumers make decisions requires a framework that captures the interplay between structural conditions, interpersonal experiences, and psychological processes. Drawing on minority stress theory and research on discrimination, vulnerability, and institutional trust, this framework brings together four interconnected constructs: trust, risk perception, discrimination history, and affirming cues (Hatzenbuehler, 2010; Meyer, 2003). These constructs were selected because they consistently appear across LGBTQ+ disparities research as central mechanisms shaping how individuals interpret and respond to service environments.

Each construct plays a distinct role. Trust influences whether consumers feel comfortable engaging with providers or institutions. Risk perception shapes how individuals assess the potential for harm or mistreatment. Discrimination history provides the experiential foundation that informs expectations and shapes future behavior. And affirming cues serve as signals, sometimes subtle, sometimes explicit, that help LGBTQ+ consumers determine whether a service environment is safe and respectful.

Together, these constructs help explain how LGBTQ+ individuals assess safety, anticipate treatment, and decide whether, when, and how to engage with services across health care, housing, financial institutions, and daily consumer settings. The sections that follow define each construct in more detail and describe how they interact to shape LGBTQ+ consumer decision-making.

### Trust as a foundation of LGBTQ+ consumer decision-making

Trust sits at the heart of most consumer interactions, shaping whether people feel comfortable seeking services, sharing personal information, following recommendations, or returning (Whitehead et al., 2016). For LGBTQ+ consumers, however, trust is often more fragile and harder to establish. For example, many have experienced discrimination or disrespect in past interactions, which can linger and influence how they approach new service environments (Kcomt, 2019; Meyer, 2003). In health care, for example, LGBTQ+ patients frequently report being misgendered, dismissed, or denied care altogether. These are experiences that understandably erode trust and lead some to delay or avoid seeking services (Puckett et al., 2018; Whitehead et al., 2016). Similar patterns appear in financial services, in which LGBTQ+ adults report higher rates of discrimination when applying for credit, seeking financial advice, or navigating banking systems (Casey et al., 2019; Human Rights Campaign Foundation, 2024). Housing interactions can also be fraught, with LGBTQ+ renters and buyers encountering subtle or overt forms of bias from landlords, neighbors, or real estate professionals (Romero et al., 2020).

It helps to distinguish between two types of trust: institutional and interpersonal. Institutional trust refers to confidence in systems, including health care, housing markets, financial institutions, and government agencies. LGBTQ+ consumers often approach these systems with caution. Historical pathologization of LGBTQ+ identities, discriminatory lending or housing practices, and inconsistent legal protections all contribute to lower levels of institutional trust (Hatzenbuehler, 2010; Romero et al., 2020). Interpersonal trust, on the other hand, focuses on individual providers or service workers. LGBTQ+ consumers often gage interpersonal trust through cues, such as communication style, respect for pronouns, nonjudgmental attitudes, and demonstrated cultural competence (Cahill and Makadon, 2014; Whitehead et al., 2016).

Trust also interacts closely with risk perception. For example, when LGBTQ+ consumers anticipate a high likelihood of discrimination or harm, trust becomes more difficult to build even in environments that may otherwise appear welcoming. This interplay between trust and perceived risk is central to understanding how LGBTQ+ individuals navigate consumer settings.

### Risk perception and LGBTQ+ consumer behavior

Risk perception plays a significant role in shaping LGBTQ+ consumer decisions. Many LGBTQ+ individuals approach service environments with a heightened awareness of potential harm, informed by both personal experiences and broader patterns of discrimination. This sense of risk can influence decisions ranging from whether they seek services to how openly they communicate once they do.

In health care, for instance, transgender and gender-diverse people often report delaying or avoiding care because they anticipate being misgendered, dismissed, or denied treatment (Kcomt, 2019; Puckett et al., 2018). In housing, LGBTQ+ renters may worry about discriminatory treatment from landlords or neighbors, prompting them to limit their housing searches to areas perceived as safer or more inclusive (Romero et al., 2020). These concerns are not hypothetical. Rather, they reflect well-documented patterns of discrimination across multiple areas.

LGBTQ+ consumers weigh several types of risk simultaneously. Identity-based risk involves the possibility of being outed, misgendered, or judged. Safety risk concerns the potential for harassment or violence. Privacy risk involves the fear that sensitive information may be mishandled or disclosed without consent. And financial risk includes concerns about being denied credit, charged higher rates, or treated unfairly in financial transactions. These risks, or combinations thereof, are grounded in real experiences of discrimination across health care, housing, employment, and financial services (Center for American Progress, 2025; Kaiser Family Foundation, 2024).

In addition, high perceived risk can undermine trust, even when providers or institutions attempt to signal inclusivity. To navigate these challenges, LGBTQ+ consumers often rely on strategies such as seeking LGBTQ-specific or LGBTQ-friendly providers, turning to community recommendations, conducting extensive online research, avoiding services perceived as hostile, or using digital platforms that allow for greater anonymity.

### Discrimination history as a determinant of consumer choice

Past experiences with discrimination shape LGBTQ+ consumer behavior in powerful ways. These experiences can occur in health care settings, housing markets, workplaces, financial institutions, retail environments, or public accommodations. For transgender and gender-diverse individuals, discrimination in health care is particularly well documented, including refusal to treat such individuals, verbal harassment, and providers’ lack of knowledge about gender-affirming care (Kcomt, 2019; Puckett et al., 2018). These encounters often lead to avoidance of care and unmet health needs.

Housing discrimination is also a persistent issue. For example, research and policy reports show that LGBTQ+ people experience higher rates of housing instability and homelessness, often tied to family rejection, discriminatory treatment by landlords, and gaps in legal protections (Durso and Gates, 2012; Romero et al., 2020). These experiences can shape how LGBTQ+ consumers approach future housing searches, influencing where they look, who they trust, and how openly they disclose their identities.

Moreover, discrimination history affects both trust and risk perception. For example, individuals who have experienced discrimination may enter new service environments with heightened caution, anticipating similar treatment. Anticipated discrimination, or the fear of future mistreatment based on past experiences, can be just as influential as actual discrimination in shaping behavior (Hatzenbuehler, 2010; Meyer, 2003). These effects are often magnified for LGBTQ+ people who also belong to other marginalized groups, such as people of color, immigrants, or individuals with disabilities, who may face multiple, intersecting forms of discrimination.

### Affirming cues as signals of safety and inclusion

Affirming cues are the signals, both subtle and explicit, that help LGBTQ+ consumers determine whether a service environment is safe, respectful, and inclusive. These cues can take many forms. In health care, for example, they might include visible nondiscrimination policies, gender-neutral restrooms, inclusive intake forms, or staff who introduce themselves with their pronouns (Cahill and Makadon, 2014). In housing and financial services, affirming cues may appear in the form of explicit nondiscrimination statements, staff training in LGBTQ+ cultural competence, or marketing materials that visibly include LGBTQ+ people (Human Rights Campaign Foundation, 2024).

These cues function as heuristics. Namely, they can serve as mental shortcuts that help LGBTQ+ consumers quickly assess whether they are likely to be treated with respect. When affirming cues are present, consumers may feel more comfortable engaging with services, disclosing relevant information, and forming ongoing relationships with providers (Whitehead et al., 2016). However, affirming cues are not always reliable indicators of deeper institutional competence. For example, in some cases, organizations may display surface-level signals of inclusion without having the policies, training, or accountability structures needed to meaningfully support LGBTQ+ consumers. When affirming cues are absent, LGBTQ+ individuals may assume the environment is not inclusive and either withhold information or avoid the service altogether.

## Discussion

The purpose of this article was to develop a conceptual framework for understanding LGBTQ+ consumer decision-making across health care, housing, financial services, and everyday service environments. Drawing upon Minority Stress Theory and Intersectionality Theory, the framework integrates four key constructs: trust, risk perception, discrimination history, and affirming cues. By bringing these factors together, the framework provides a foundation for understanding how structural conditions and lived experiences influence LGBTQ+ consumers’ engagement with services and institutions.

Although foundational studies remain central to understanding minority stress, discrimination, and LGBTQ+ well-being, recent reports and survey findings continue to document persistent disparities in health care access, housing stability, financial well-being, and service experiences among LGBTQ+ populations. These contemporary findings suggest that the issues addressed by the proposed framework remain highly relevant and warrant continued scholarly attention.

The four constructs of trust, risk perception, discrimination history, and affirming cues can be incorporated in a conceptual model that explains how LGBTQ+ consumers navigate service environments. Each construct plays a distinct role, but their influence is interconnected. For example, discrimination history forms the experiential backdrop that shapes both trust and perceived risk. Individuals who have experienced discrimination in the past often enter new service settings with heightened caution, which can lower trust and increase the sense of potential danger or harm.

Trust, in turn, influences whether LGBTQ+ consumers feel comfortable engaging with providers, disclosing personal information, or returning for future services. Risk perception mediates the relationship between discrimination history and consumer behavior by shaping how individuals interpret the likelihood and severity of potential negative experiences. Affirming cues moderate these dynamics by signaling whether a service environment is likely to be safe, respectful, and inclusive. When affirming cues are present and credible, they can soften the impact of past discrimination and reduce perceived risk; when absent, they may reinforce caution or avoidance.

These relationships help explain how LGBTQ+ consumers make decisions across health care, housing, financial services, and daily service interactions (Casey et al., 2019; Whitehead et al., 2016). This framework highlights the importance of both structural conditions and interpersonal interactions, showing how past experiences, present cues, and anticipated outcomes shape consumer behavior in complex, interrelated ways.

The conceptual framework offers a way to understand the layered and often challenging experiences LGBTQ+ consumers face across multiple service areas. By bringing together trust, risk perception, discrimination history, and affirming cues, the framework highlights how structural inequities and interpersonal interactions help shape consumer decision-making. These constructs explain why LGBTQ+ individuals may approach certain services with caution, why they may rely heavily on community recommendations, and why seemingly small signals of inclusion can have such a meaningful impact.

The framework also underscores the importance of addressing both systemic and interpersonal factors. Structural inequities such as gaps in nondiscrimination protections, inconsistent enforcement of existing laws, and historical patterns of exclusion shape the broader environment in which LGBTQ+ consumers make decisions. At the same time, daily interactions with providers, staff, and service workers can either reinforce or counteract these structural forces. Understanding how these layers interact can help service providers, policymakers, and researchers develop more effective strategies for improving LGBTQ+ consumer experiences. As a conceptual framework, the model proposed here has not yet been empirically tested and should be considered a foundation for future research rather than a definitive explanation of LGBTQ+ consumer decision-making.

### Implications for service providers

Service providers play a central role in shaping LGBTQ+ consumers’ experiences. Building trust requires creating environments in which LGBTQ+ individuals feel respected, safe, and valued. Providers can accomplish this through visible nondiscrimination policies, inclusive language, culturally competent staff training, transparent complaint processes, and clear privacy protections. As services increasingly move online, organizations should also ensure that digital platforms incorporate affirming features and safeguard sensitive information. These efforts can reduce perceived risk, strengthen trust, and improve access to services for LGBTQ+ consumers.

### Implications for policymakers

Policymakers play a critical role in shaping the structural conditions that influence LGBTQ+ consumer experiences. Strengthening and consistently enforcing nondiscrimination protections across health care, housing, and financial services can help reduce barriers to access and improve consumer well-being. Policymakers can also support LGBTQ+ cultural competence training, expand funding for community-based organizations, and encourage more robust data collection efforts that identify disparities and inform policy development. These actions can help create more equitable consumer environments and reduce the structural risks LGBTQ+ individuals often face when accessing essential services.

### Implications for researchers

The framework presented provides several opportunities for future research. Empirical studies are needed to examine the relationships among trust, risk perception, discrimination history, and affirming cues across different service contexts. Researchers should also investigate how these relationships vary across LGBTQ+ subpopulations and intersecting identities. Additional work is needed to develop validated measures of LGBTQ+ consumer trust and affirming cues, as well as to explore how digital platforms and technology-mediated services influence consumer experiences. Such research can strengthen the framework and inform future efforts to improve service delivery and consumer outcomes.

## Conclusion

This article proposes a conceptual framework for understanding LGBTQ+ consumer decision-making across health care, housing, financial services, and other consumer settings. Drawing on Minority Stress Theory and Intersectionality Theory, the framework identifies trust, risk perception, discrimination history, and affirming cues as interconnected factors that influence how LGBTQ+ consumers assess service environments and make decisions. The framework highlights the ways in which structural inequalities, prior experiences, and contextual signals shape consumer behavior and access to resources. By integrating insights from multiple disciplines, this conceptual model contributes to a growing understanding of LGBTQ+ consumer experiences and provides a foundation for future empirical research, service innovation, and policy development aimed at fostering more inclusive consumer environments.
